# Fluoride-resistant *Streptococcus mutans* within cross-kingdom biofilms support *Candida albicans* growth under fluoride and attenuate the *in vitro* anti-caries effect of fluorine

**DOI:** 10.3389/fmicb.2024.1399525

**Published:** 2024-07-05

**Authors:** Yan Sun, Yanhan Chen, Qian Du, Jin Zhang, Muxin Xu, Gaozhe Zheng, Wen Zhou, Xinxuan Zhou, Lili Qiu, Yihuai Pan, Keke Zhang

**Affiliations:** ^1^School and Hospital of Stomatology, Wenzhou Medical University, Wenzhou, China; ^2^Stomatology Hospital, School of Stomatology, Zhejiang University School of Medicine, Zhejiang Provincial Clinical Research Center for Oral Diseases, Key Laboratory of Oral Biomedical Research of Zhejiang Province, Cancer Center of Zhejiang University, Hangzhou, China; ^3^State Key Laboratory of Oral Diseases, National Clinical Research Center for Oral Diseases, West China Hospital of Stomatology, Sichuan University, Chengdu, China; ^4^Department of Cariology and Endodontics, West China Hospital of Stomatology, Sichuan University, Chengdu, China; ^5^Fujian Key Laboratory of Oral Diseases, Fujian Provincial Engineering Research Center of Oral Biomaterial, Stomatological Key Lab of Fujian College and University, School and Hospital of Stomatology, Fujian Medical University, Fuzhou, China

**Keywords:** fluoride-resistant strain, *Streptococcus mutans*, *Candida albicans*, cross-kingdom biofilm, caries, fluorine

## Abstract

Fluoride-resistant *Streptococcus mutans* (*S. mutans*) might affect the ecological balance of biofilms in the presence of fluoride. We used a *S. mutans* and *Candida albicans* (*C. albicans*) cross-kingdom biofilm model to investigate whether fluoride-resistant *S. mutans* in biofilms would support *C. albicans* growth under fluoride stress and attenuate the *in vitro* anti-caries effect of fluorine. The impact of fluoride-resistant *S. mutans* on formation of cross-kingdom biofilms by *S. mutans* and *C. albicans* in the presence of fluoride was investigated *in vitro* using the crystal violet staining assay. Biofilm constitution was determined using colony-forming unit (CFU) counts and fluorescent *in situ* hybridization (FISH). Extracellular polysaccharide (EPS) generation in biofilms was determined by EPS/bacterial dying and water-insoluble polysaccharide detection. Acid production and demineralization were monitored using pH, lactic acid content, and transversal microradiography (TMR). The gene expression of microorganisms in the cross-kingdom biofilm was measured using qRT-PCR. Our results showed that both *C. albicans* and fluoride-resistant *S. mutans* grew vigorously, forming robust cross-kingdom biofilms, even in the presence of sodium fluoride (NaF). Moreover, fluoride-resistant *S. mutans*-containing cross-kingdom biofilms had considerable cariogenic potential for EPS synthesis, acid production, and demineralization ability in the presence of NaF than fluoride-sensitive *S. mutans*-containing biofilms. Furthermore, the gene expression of microorganisms in the two cross-kingdom biofilms changed dissimilarly in the presence of NaF. In summary, fluoride-resistant *S. mutans* in cross-kingdom biofilms supported *C. albicans* growth under fluoride and might attenuate the anti-caries potential of fluorine by maintaining robust cross-kingdom biofilm formation and cariogenic virulence expression *in vitro* in the presence of NaF.

## Introduction

Dental caries, one of the most widespread biofilm-dependent diseases that plague people's health globally, is caused by biofilm dysbiosis (Cheng et al., 2022). *Streptococcus mutans* (*S. mutans*) and *Candida albicans* (*C. albicans*) played a crucial role in cariogenic biofilm formation for dental plaque (Guo et al., 2023). *S. mutans* is an opportunistic cariogenic microorganism found in biofilms. It produces extracellular polysaccharide (EPS)-rich biofilm extracellular polymeric substances (biofilm matrix), which significantly contribute to adhesion, biofilm formation, maintenance, and cariogenicity (Hajishengallis et al., 2017). Moreover, it generates organic acids to promote dental caries, while displaying acid tolerance to respond to acid stress. *C. albicans*, oral resident yeast, is closely connected with different caries such as root caries and early childhood caries (ECC) (Du et al., 2022). Interestingly, it is frequently detected along with *S. mutans* in carious lesions, peculiarly in children who suffer from ECC, an aggressive form of caries that affects children under 6 years (Du et al., 2022).

Further study showed that though an interaction between *S. mutans* and *C. albicans* could be antagonistic in some case, they work together to promote caries (Kim et al., 2021; Lu et al., 2023). The appearance of one would boost the other in the cross-kingdom biofilm, mainly through glucosyltransferase B (GtfB, encoded by *gtfB*) of *S. mutans*, metabolism, and signaling processes (Chan et al., 2020). *S. mutans* exoenzyme GtfB, the expression of which could be strengthened by *C. albicans*, binds to the fungal surface and synthesizes extracellular polysaccharides (EPS) in the presence of sucrose (Hwang et al., 2017; Kim et al., 2018). The EPS on the *C. albicans* surface could further improve co-adhesion, cross-kingdom biofilm development, and antifungal drug tolerance of *C. albicans* (Kim et al., 2018). In addition, glucose and fructose formed after breakdown of sucrose by *S. mutans* can be utilized by *C. albicans*, and enhanced fructose metabolism in *C. albicans* facilitates hyphal morphogenesis (Ellepola et al., 2019). The acid products from carbohydrate metabolism result in an acidic environment, favoring the growth of these two acidogenic and acid-tolerant microorganisms in cariogenic biofilms (Kim et al., 2017). Moreover, *C. albicans* synthesizes proteinases that can destroy collagen in dentin caries, promoting its invasion into dentinal tubules under acidic environments, and farnesol that heightens biofilm development, *gtfBC* expression, and Gtf activity of *S. mutans* (Kim et al., 2017; Lobo et al., 2019). Furthermore, sigX, an alternative sigma factor induced by quorum sensing signals in *S. mutans*, was strongly induced in bacterial–fungal biofilms (Sztajer et al., 2014). These synergistic interactions explained that coculture or coinfection of *S. mutans* together with *C. albicans* produced more EPS, and the biomass showed stronger cariogenicity than the single species *in vitro* and *in vivo* (Falsetta et al., 2014; Sampaio et al., 2019; Khoury et al., 2020).

Fluoride is an effective and commonly used tactic for caries prevention, including ECC. The anti-caries effects of fluoride occur mainly through decreasing enamel demineralization and increasing enamel remineralization (Pitts et al., 2017). In addition, high fluoride levels have bacteriostatic and bactericidal effects on oral organisms (Pitts et al., 2017). The antimicrobial effects of fluoride are not only based on its fluence on microbial enzymic activity by binding directly in forms of fluorion or hydrogen fluoride, and forming metal–fluoride complexes, but also serving as transmembrane proton carriers in the form of hydrogen fluoride to bring protons into the cell, dissipating the proton gradient (Koo, 2008). However, the wide application of fluoride would give rise to fluoride-resistant oral bacteria such as *S. mutans*, and its clinical isolates have been reported (Streckfuss et al., 1980; Elhage et al., 2022). Most stable fluoride-resistant *S. mutans* were derived from induction *in vitro*, which was regrown in media with higher fluoride concentrations than that can be tolerated by fluoride-sensitive (wild-type) strains after 50 generations of subculture in fluoride-free media (Liao et al., 2017). The isolated fluoride-resistant *S. mutans* could have changed its colonial morphology, reduced growth velocity, enhanced acid tolerance, and biofilm formation ability (Zhu et al., 2012; Liao et al., 2015; Cai et al., 2017). The genes *smu.1289c* (also named *eriC1* or *perB*), *cas3*, and *frtR* are related to the intrinsic fluoride tolerance of *S. mutans*. Deletion of these genes resulted in decreased fluoride resistance, except for *smu.1290c* (also named *eriC1a* or *perA*), which is controversial (Men et al., 2016; Murata and Hanada, 2016; Tang et al., 2019; Lu et al., 2020). Some genes are closely related to the high fluoride resistance of *S. mutans*. A nucleotide variation within the *mutp* promoter of *S. mutans* upregulated *mut, smu.1290c*, and *smu.1289c*, thus enhancing bacterial fluoride resistance (Liao et al., 2016, 2018).

Our previous study showed that fluoride-resistant *S. mutans* achieved a competitive advantage over antagonistic *Streptococcus sanguinis* (*S. sanguinis*) and displayed stronger cariogenic potential in competitive biofilms formed in the presence of NaF (Zhang K. et al., 2022). However, no reports have focused on the effect of fluoride-resistant *S. mutans* on cross-kingdom biofilms under the influence of fluoride. Will fluoride-resistant *S. mutans* support *C. albicans* growth under fluoride stress and resist the anti-biofilm effect of fluoride, thus limiting the anti-caries effect of fluoride? We proposed this study to answer this question.

## Materials and methods

### Microorganisms and growth condition

All microorganisms used in this study were obtained from the School and Hospital of Stomatology, Wenzhou Medical University. *C. albicans* SC 5314, *S. mutans* UA159 (wild-type strain, named as fluoride-sensitive *S. mutans* blow), and its induced fluoride-resistant strain were used throughout the study. Stable fluoride-resistant *S. mutans* strains were obtained using previously described procedures (Zhang K. et al., 2022). Briefly, overnight-cultured *S. mutans* were spread on a brain–heart infusion (BHI; Oxoid; Basingstoke, UK) agar plate supplemented with 0.5 g/L NaF. A single clone of *S. mutans* grown on the BHI agar plate with 0.5 g/L NaF was subcultured 50 times on BHI agar plates without fluoride for subculturing to obtain fluoride-resistant *S. mutans*.

Overnight-cultured *S. mutans, C. albicans*, and fluoride-resistant *S. mutans* in tryptone-yeast extract (TYE) broth containing 1% glucose were used for microbial proliferation. The final inoculum for single-species biofilm formation was 10^6^ colony-forming unit (CFU)/mL fluoride-sensitive or fluoride-resistant *S. mutans* or 10^4^ CFU/mL *C. albicans* in TYE broth containing 1% sucrose. The final inoculum for cross-kingdom biofilm formation was 10^6^ CFU/mL fluoride-sensitive or fluoride-resistant *S. mutans* and 10^4^ CFU/mL *C. albicans* in TYE broth containing 1% sucrose. All biofilms were formed in 96- or 24-well plates for 24 h, unless otherwise stated. The total inoculum volume for biofilms formed in 96-well plates was 200 μL. For biofilms formed in 24-well plates, a glass sheet was placed inside beforehand, and the total inoculum volume was 2 mL. NaF was added at the beginning of biofilm formation in all experiments, and the final NaF concentrations were 0, 275 (fluoride concentration within regular toothpaste after dilution at 1:3), and 1,250 mg/L (fluoride concentration in the prescribed toothpaste after dilution at 1:3) (Zhang K. et al., 2022). In all experiments, the microorganisms were grown at 37°C and 5% CO_2_ (v/v).

### Crystal violet assay

A crystal violet assay was conducted to assess biofilm formation (Sun et al., 2019). Accordingly, 24-h single-species and cross-kingdom biofilms formed in 96-well plates were fixed with methanol and stained with 0.1% crystal violet. Representative crystal violet-stained biofilms were observed by using a stereomicroscope (Nikon SMZ800, Nikon Corporation, Japan). The stained crystal violet in biofilms was dissolved using 33% acetic acid for quantitative testing by recording the OD_590nm_ by using a microplate reader (SpectraMax M5, Molecular Devices, USA).

### Colony-forming unit counts

The 24-h cross-kingdom biofilms formed in 24-well plates were collected by scraping with sterilized blades and sonication/vortexing in phosphate-buffered saline (PBS). After serial dilution in PBS, BHI agar and Sabouraud's dextrose Agar (SDA, Oxoid) were used to support the growth of total microorganisms and *C. albicans*, respectively (Zhang et al., 2016).

### Fluorescent *in situ* hybridization

FISH was used to monitor the inter-kingdom biofilm constitution. Briefly, the 24-h biofilms in 24-well plates were fixed with 4% paraformaldehyde, lysed with lysozyme, and dehydrated using gradient ethanol (50%, 80%, and 96%). Then, *S. mutans* and *C. albicans* were stained with species-specific fluorescent probes in a hybridization solution containing 20% formamide (Kempf et al., 2000; Sun et al., 2019). The probe details are presented in Supplementary Table 1. Random biofilms were imaged with a Nikon confocal laser scanning microscope (CLSM, Nikon A1, Nikon Corporation, Japan) equipped with a 60 × oil immersion objective lens.

### Polysaccharide measurement

The water-insoluble glucan content of biofilms was determined using the anthrone method, and polysaccharide staining was used to analyze the polysaccharide distribution in biofilms (Zhang L. et al., 2022). For the anthrone method, 24-h biofilms formed in 24-well plates were collected, washed with deionized water, and resuspended in 0.4 mol/L NaOH. After centrifugation, the collected supernatant was incubated with 1:3 (v/v) anthrone reagent at 95°C for 6 min. The OD_625nm_ was monitored by using a microplate reader (SpectraMax M5). The water-insoluble glucan content of biofilms was computed based on standard curves (Supplementary Figure 1).

For polysaccharide observation, dextran, α-D-glucopyranose polysaccharides, and total microorganisms were stained using 2.5 μM Alexa Fluor 647-labeled dextran conjugate (Molecular Probes, Invitrogen Corp., Carlsbad, CA, USA), 100 μg/mL concanavalin A conjugate (Molecular Probes), and 2.5 μM SYTO 9 (Molecular Probes, Invitrogen Corp., Carlsbad, CA, USA), respectively, to analyze the EPS (Adav et al., 2010). Alexa Fluor 647-labeled dextran conjugate (excitation/emission wavelength, 650/668 nm, respectively) was dissolved in the inoculum for biofilm formation in 24-well plates, whereas concanavalin A conjugate (excitation/emission wavelength, 555/580 nm, respectively) and SYTO 9 (excitation/emission wavelength, 480/500 nm, respectively) were used to stain for 30 min after biofilm culture. Biofilms were imaged utilizing a Nikon CLSM equipped with an oil immersion objective lens at 60×.

### pH and lactic acid content measurement

The pH of the supernatants in 24-well plates 24 h after biofilm formation was measured using Orion Dual Star, pH/ISE Benchtop (Thermo Scientific; Waltham, MA, USA).

For lactic acid content measurement, the protocol was used as previously described (Zhang et al., 2015). After washing with cysteine peptone water (CPW), 24-h biofilms formed in 24-well plates were cultured with buffered peptone water (BPW) containing 0.2% sucrose for 3 h. OD_340nm_ was detected before and after incubation by using a microplate reader (SpectraMax M5). The amount of lactic acid produced by the biofilm was calculated using a standard curve (Supplementary Figure 2).

### Demineralization

This study was approved by the ethics committee of the School and Hospital of Stomatology, Wenzhou Medical University (ethical codes: WYKQ2020006). The demineralization ability of biofilms was determined by a human tooth demineralization model. Extracted human teeth were obtained from the Department of Oral and Maxillofacial Surgery and stored in 0.05% thymol solution, as described previously (Du et al., 2021). The labial crown of the isolated tooth was trimmed to obtain 5 × 5 × 3 mm^3^ sections and wrapped in polymethylmethacrylate. After coating with an acid-resistant varnish, a standardized enamel surface window (4 × 4 mm^2^) was allowed to be demineralized by the biofilm. The enamel surface was polished with sandpaper (800–2,500 grit), followed by ultrasonic treatment for washing. Totally, 3.6 mL 10^6^ CFU/mL fluoride-sensitive or -resistant *S. mutans* and 10^4^ CFU/mL *C. albicans* in TYE broth containing 1% sucrose was seeded in each well of a 12-well plate with different NaF concentrations. Then, 1.8 mL of the medium was changed every 24 h for 5 days. Later, the biofilm was removed, and enamel slices were prepared. Mineral loss by biofilms was detected using transversal microradiography (TMR).

### RNA isolation and real-time PCR

Twenty-four-h biofilms formed in 10-cm Petri dishes with 10 mL media containing different NaF concentrations were collected to isolate RNA. TRIzol reagent (Invitrogen, Carlsbad, CA, USA) was used as previously described, using glass beads to break the cell wall in the Precellys 24 system (Bertin Technologies; Paris, France) (Zhang K. et al., 2022). RNA concentration and purity were measured using a NanoDrop 2000 spectrophotometer, and integrality was verified by electrophoresis. Reverse transcription was performed using a PrimeScript™ RT Reagent Kit with gDNA Eraser (Takara; Tokyo, Japan) according to the manufacturer's instructions.

TB Green^®^ Premix Ex Taq™ II (Tli RNaseH Plus, Takara) was used for real-time PCR. Briefly, a 20 μL mixture comprising 10 μL 2 × TB Green Premix Ex Taq II, 10 μM forward and reverse primers, 0.4 μL 50 × ROX Reference Dye, and 1 μL DNA template was added into each well of Applied Biosystems MicroAmp^®^ Fast 8-Tube Strips, and PCR cycles were run in a StepOnePlus Real-Time PCR System (Applied Biosystems; CA, USA). The PCR procedure was set as 30 s at 95°C, 40 cycles (5 s at 95°C, 30 s at 55°C, and 30 s at 72°C). Gene expression was normalized to 16S and 18S rRNA expression for *S. mutans* and *C. albicans*, respectively, using log_2_-transformed fold change (0 mg/mL NaF groups were set as corresponding control respectively). The primers used are listed in Supplementary Table 2.

### Statistical analysis

All assays were conducted in triplicate, individually. Significant effects of the variables were distinguished by one-way ANOVA, followed by SNK. Differences were considered significant if *P* < 0.05. Statistical analysis was performed utilizing software SPSS16.0 (SPSS Inc.; Chicago, IL, USA).

## Results

### Fluoride-resistant *S. mutans*-associated biofilms showed stronger biofilm formation ability with NaF treatment

**Figure 1:**
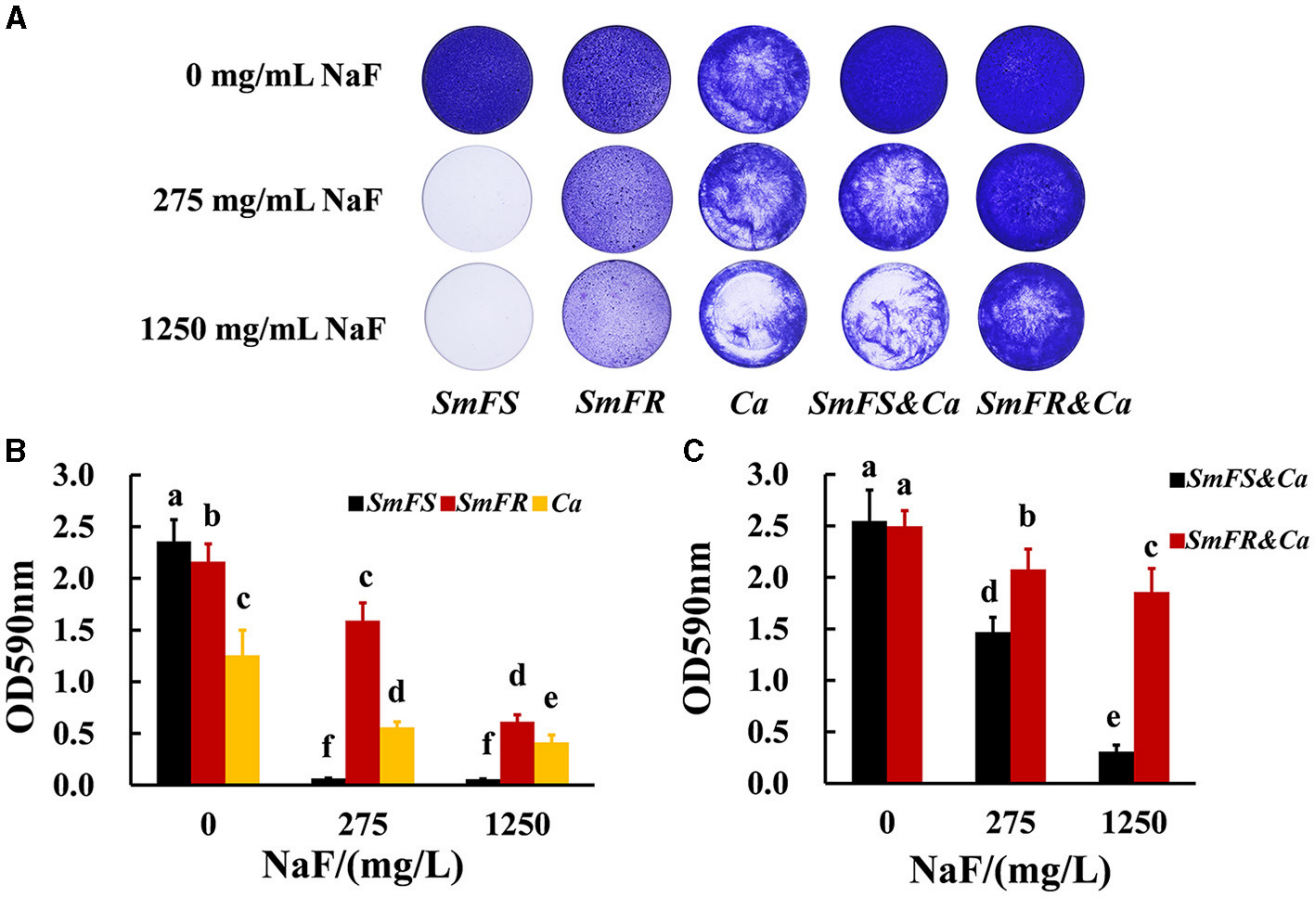
Biomass of biofilms revealed by crystal violet staining assay. **(A)** Representative images of crystal violet-dyed biofilms; **(B)** quantitative results of single-species biofilms based on crystal violet staining; **(C)** quantitative results of cross-kingdom biofilms based on crystal violet staining (*SmFS* represented fluoride-sensitive *S. mutans, SmFR* represented fluoride-resistant *S. mutans*, and *Ca* represented *C. albicans*. Data were presented as mean ± standard deviation. Values with dissimilar letters are identified as significantly different from each other, *P* < 0.05).

Fluoride-resistant *S. mutans* could form biofilms even in the presence of 1,250 mg/L NaF, whereas fluoride-sensitive *S. mutans* scarcely formed biofilms even at 275 mg/L NaF (Figure 1A). Although NaF inhibited *C. albicans* biofilm formation, *C. albicans* formed biofilms in the presence of 1,250 mg/L NaF (Figure 1A). Fluoride-resistant *S. mutans*-containing cross-kingdom biofilms were more resistant to fluoride than fluoride-sensitive *S. mutans*-containing cross-kingdom biofilms. The quantitative results (Figures 1B, C) showed an analogous tendency in comparison to the results shown in Figure 1A. Fluoride-resistant *S. mutans*-containing cross-kingdom biofilms showed a prominently higher OD values than that of fluoride-sensitive *S. mutans*-containing cross-kingdom biofilms under NaF stress (*P* < 0.05; Figure 1).

### Both fluoride-resistant *S. mutans* and *C. albicans* in cross-kingdom biofilms benefited from fluoride resistance and grew better in the presence of NaF

**Figure 2:**
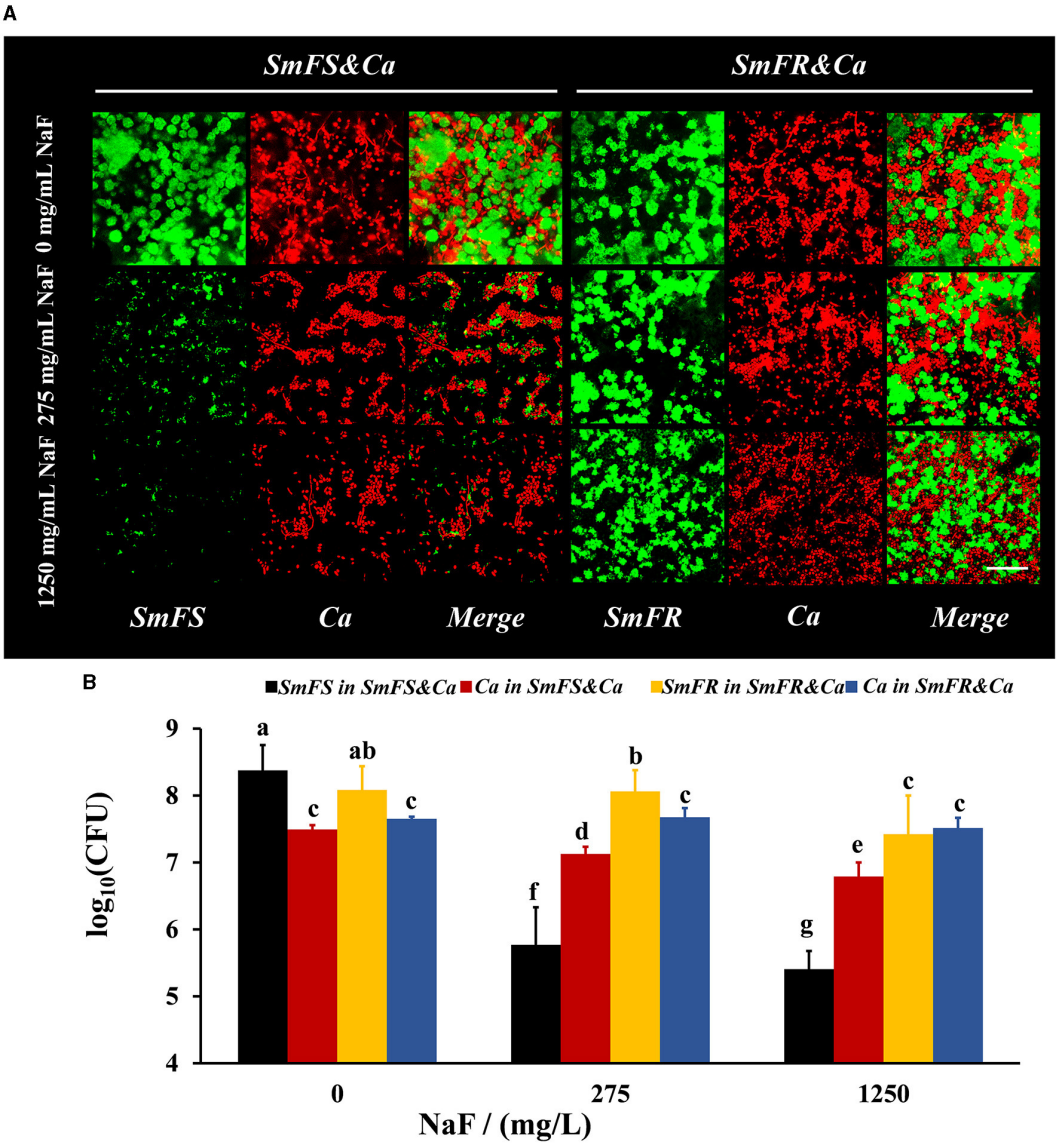
Composition of cross-kingdom biofilms. **(A)** FISH of cross-kingdom biofilms. *S. mutans* were stained green, and *C. albicans* were stained red, scale bar = 50 μm; **(B)** CFU of cross-kingdom biofilms (*SmFS* represented fluoride-sensitive *S. mutans, SmFR* represented fluoride-resistant *S. mutans*, and *Ca* represented *C. albicans*. Data were presented as mean ± standard deviation. Values with dissimilar letters are identified as significantly different from each other, *P* < 0.05).

FISH results showed that both fluoride-resistant *S. mutans* and *C. albicans* benefited from the fluoride-resistant strain in the presence of NaF (Figure 2A). Both *C. albicans* and *S. mutans* grew vigorously in the two cross-kingdom biofilms in the absence of NaF. However, the microbial growth in the fluoride-sensitive *S. mutans*-containing biofilms, particularly that of *S. mutans*, was limited in the presence of NaF. Moreover, the fluoride-resistant *S. mutans* and *C. albicans* were not affected significantly as fluoride-sensitive *S. mutans-*containing ones and formed robust cross-kingdom biofilms, even in the presence of 1,250 mg/L NaF (Figure 2A). According to colony counts in fluoride-sensitive *S. mutans*-containing cross-kingdom biofilms, *S. mutans* count decreased by approximately 2.6 and 3.0 log10 CFU in the presence of 275 and 1,250 mg/L NaF, respectively, while *C. albicans* count reduced by approximately 57% and 80%, respectively, compared to in the absence of NaF (Figure 2B). However, fluoride-resistant *S. mutans* count in fluoride-resistant *S. mutans*-containing cross-kingdom biofilms diminished by 0.02 and 0.66 log10 CFU in the presence of 275 and 1,250 mg/L NaF, respectively, than in the absence of NaF, and *C. albicans* count did not change significantly (*P* > 0.05; Figure 2B).

### Fluoride-resistant *S. mutans*-containing cross-kingdom biofilms produced more EPS in the presence of NaF

**Figure 3:**
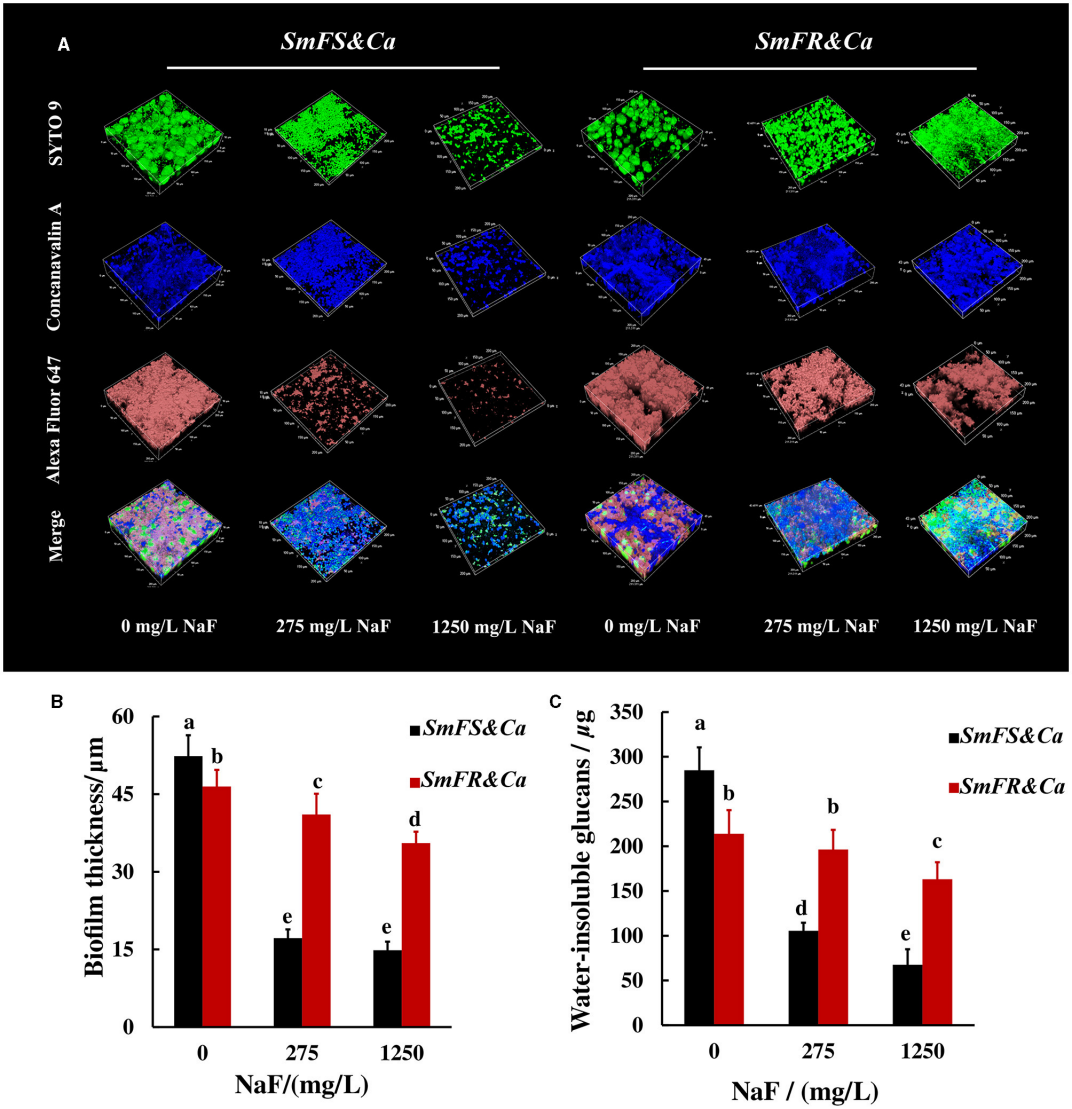
EPS of cross-kingdom biofilms. **(A)** 3D reconstruction of biofilms, the horizontal extent was 200 × 200 μm^2^, dextran (worked as an acceptor and was incorporated into glucan) together with α-D-glucopyranose, and total microorganisms were stained by Alexa Fluor 647, concanavalin A, and SYTO 9, respectively; **(B)** biofilm thickness of cross-kingdom biofilms; **(C)** insoluble exopolysaccharides of cross-kingdom biofilms (*SmFS* represented fluoride-sensitive *S. mutans, SmFR* represented fluoride-resistant *S. mutans*, and *Ca* represented *C. albicans*. Data were presented as mean ± standard deviation. Values with dissimilar letters are identified as significantly different from other, *P* < 0.05).

The reconstructed 3D biofilm showed that the presence of microorganisms or EPS reduced substantially after growing *S. mutans*-containing biofilms in the presence of NaF. Fluoride-resistant *S. mutans-*containing cross-kingdom biofilms were more resistant to NaF, with more microorganisms and higher EPS content, than fluoride-sensitive *S. mutans*-containing biofilms in the presence of NaF (Figure 3A). Reconstructed 3D fluoride-resistant *S. mutans*-containing biofilms were thicker in the presence of NaF stress (*P* < 0.05; Figure 3B). The anthrone method results showed that fluoride-sensitive *S. mutans*- and *C. albicans*-containing inter-kingdom biofilms had more and less water-insoluble glucans than fluoride-resistant *S. mutans*- and *C. albicans*-containing inter-kingdom biofilms in the absence and presence of NaF, respectively (*P* < 0.05; Figure 3C).

### Fluoride-resistant *S. mutans*-containing cross-kingdom biofilms generated more acid and displayed stronger demineralization effect in the presence of NaF

**Figure 4:**
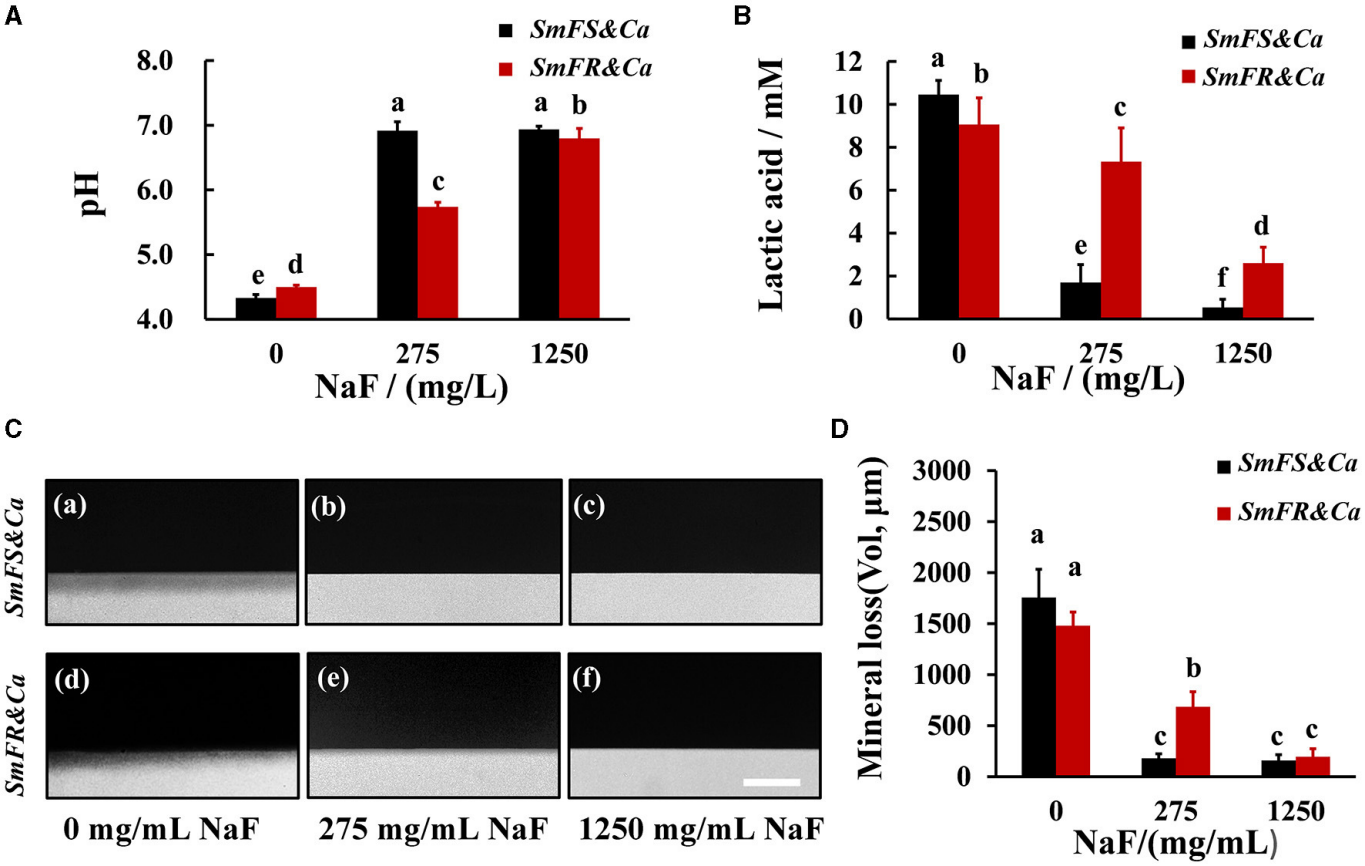
Acid production and demineralization of biofilms. **(A)** pH of the supernatant pH of 24-h biofilms; **(B)** lactic acid production of cross-kingdom biofilms; **(C)** representative demineralization images caused by biofilms revealed by TMR in fluoride-sensitive *S. mutans*-containing cross-kingdom biofilms (a–c), fluoride-resistant *S. mutans*-containing cross-kingdom biofilm (d–f), bar = 100 μm; **(D)** mineral loss of human tooth enamel caused by biofilms based on TMR (*SmFS* represented fluoride-sensitive *S. mutans, SmFR* represented fluoride-resistant *S. mutans*, and *Ca* represented *C. albicans*. Data were presented as mean ± standard deviation. Values with dissimilar letters are identified as significantly different from other, *P* < 0.05).

The degree of influence of NaF on supernatant pH was different between the two cross-kingdom biofilms (Figure 4A). The pH for fluoride-sensitive *S. mutans*-containing cross-kingdom biofilms increased from approximately 4.29 to approximately 6.80 and 6.90, separately, by increasing the NaF concentration. However, the pH for fluoride-resistant *S. mutans*-containing biofilms in the presence of 275 and 1,250 mg/L NaF was lower than that for the fluoride-sensitive *S. mutans*-containing biofilms, particularly in the presence of 275 mg/L NaF (*P* < 0.05; Figure 4A). Although NaF substantially inhibited lactic acid production, fluoride-resistant *S. mutans*-containing cross-kingdom biofilms produced 4.3- and 4.8-fold more lactic acid than *S. mutans*-containing biofilms in the presence of 275 and 1,250 mg/L NaF, respectively (Figure 4B). Likewise, both qualitative (Figure 4C) and quantitative (Figure 4D) TMR results from a human tooth demineralization model showed that the fluoride-resistant or fluoride-sensitive *S. mutans*-containing cross-kingdom biofilms demineralized significantly in the absence of NaF, which was inhibited by NaF addition. However, fluoride-resistant *S. mutans*-containing biofilms still exhibited considerable demineralization effects in the presence of 275 mg/L NaF (Figures 4C(e), D).

### Microbial genes expressed differently in the two cross-kingdom biofilms in the presence of NaF

**Figure 5:**
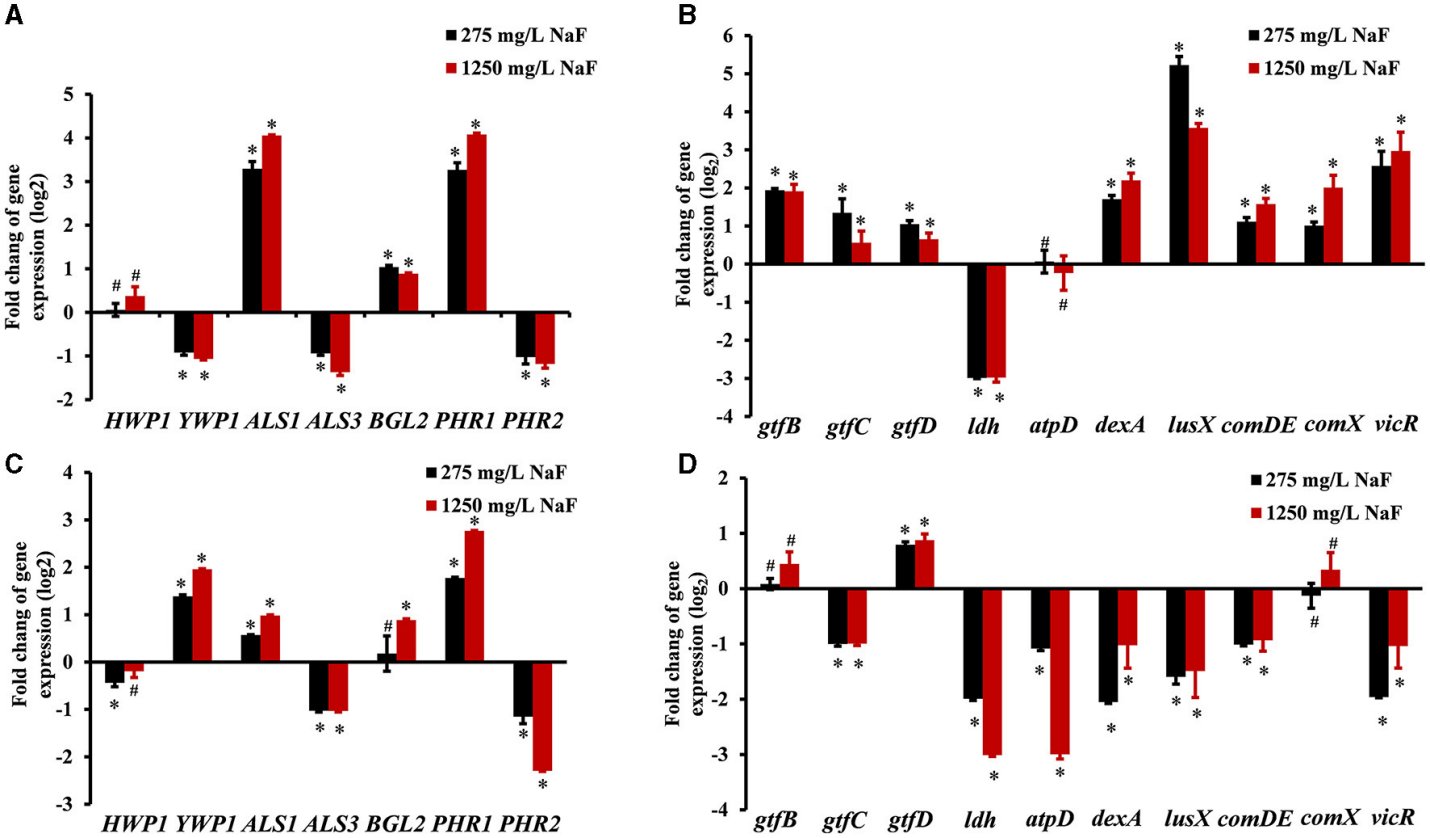
Gene expression in cross-kingdom biofilms. **(A)** gene expression of *C. albicans* in *S. mutans* fluoride-sensitive *S. mutans-*containing cross-kingdom biofilms; **(B)** gene expression of *S. mutans* fluoride-sensitive in cross-kingdom biofilms; **(C)** gene expression of *C. albicans* within fluoride-resistant *S. mutans-*containing cross-kingdom biofilms; **(D)** gene expression of fluoride-resistant *S. mutans* in cross-kingdom biofilms (Data were presented as mean ± standard deviation. # means no significant difference, *P* > 0.05, * means significant difference, *P* < 0.05).

For *C. albicans*, fungal hyphal/yeast-specific genes *HWP1* (hyphal wall protein HWP1) and *YWP1* (yeast wall protein YWP1), adhesion genes *ALS1* (human β-actin agglutinin-like sequence ALS1), and *ALS3* (agglutinin-like sequence ALS3) are closely related to its pathogenicity. *BGL2* (glucan transferase BGL2) is an EPS production gene, while *PHR1* (glycosidase PHR1) and *PHR2* (glycosidase PHR2) are vital genes for pH regulation. For *S. mutans*, EPS production, acid production, and tolerance are important cariogenic virulence factors. Thus, bacterial EPS production genes including *gtfB* (glucosyltransferase GtfB), *gtfC* (glucosyltransferase GtfC), *gtfD* (glucosyltransferase GtfD), EPS remodeling gene *dexA* (dextranase DexA), acid production gene *ldh* (lactic dehydrogenase LDH), and acid tolerance gene *atpD* (F0F1-H/F-ATPase β subunit of the F1 protein atpD) were monitored. Moreover, signaling processes are involved in the interaction between *S. mutans* and *C. albicans*, and genes *lusX* (S-ribosylhomocysteine lyase lusX), com*DE* (histidine kinase comD and response regulator comE), *comX* (alternative sigma factor SigX), and v*icR* (response regulator vicR) were also detected. The changes in microbial gene expression in the presence of NaF are shown in Figure 5. The expression of *ALS1, BGL2* (except in the fluoride-resistant *S. mutans*-containing biofilm grown in the presence of 275 mg/L NaF), and *PHR1* in *C. albicans* was upregulated significantly, while the expression of *ALS3* and *PHR2* was notably downregulated in all biofilms in the presence of NaF (*P* < 0.05). *C. albicans YWP1* expression was downregulated and upregulated in fluoride-sensitive and fluoride-resistant *S. mutans*-containing biofilms, respectively, in the presence of NaF (*P* < 0.05). In addition, *C. albicans HWP1* was not affected significantly, except in in the fluoride-resistant *S. mutans*-containing biofilm at 275 mg/mL NaF. Genes *gtfB, gtfC, gtfD, dexA, luxS, comDE, comX*, and *vicR* in *S. mutans* were significantly upregulated (*P* < 0.05); however, *ldh* was downregulated (*P* < 0.05) and *atpD* was unaffected significantly (*P* > 0.05). *gtfC, ldh, atpD, dexA, luxS, comDE*, and *vicR* were significantly downregulated in the fluoride-resistant *S. mutans*-containing biofilms (*P* < 0.05), while *gtfB* and *comX* expression was unchanged (*P* > 0.05) and *gtfD* was upregulated (*P* < 0.05).

## Discussion

We explored the effect of fluoride-resistant *S. mutans* on cross-kingdom biofilms after fluoride application to determine whether fluoride-resistant strains would support *C. albicans* growth under fluoride stress and abate the anti-caries outcome induced by fluoride. Our *in vitro* results indicate that fluoride-resistant *S. mutans* did support *C. albicans* growth under fluoride damage, which was reflected in much more fungus within cross-kingdom biofilms under NaF. In addition, it restricted the anti-biofilm effect of NaF on cross-kingdom biofilms in the following ways. First, both fluoride-resistant *S. mutans* and *C. albicans* grew substantially, forming robust cross-kingdom biofilms even with fluoride application. Moreover, fluoride-resistant *S. mutans*-containing cross-kingdom biofilms still showed considerable cariogenic potential with EPS synthesis, acid production, and demineralization in the presence of NaF than fluoride-sensitive *S. mutans*-containing biofilms. In addition, the gene expression of microbes in different cross-kingdom biofilms altered differently in the presence of NaF.

The fluoride-resistant *S. mutans* used in this study showed a sluggish growth rate without NaF compared to the fluoride-sensitive strain (Supplementary Figure 3), which was in accordance with the results of a previous study (Liao et al., 2015). This may partly explain why the fluoride-resistant strain from the single-species biofilm was not as strong as the fluoride-sensitive strain and was less abundant in the cross-kingdom biofilms without NaF. However, the survival advantage of fluoride-resistant *S. mutans* over the fluoride-sensitive strain increased with the increase in the NaF concentration. Remarkably, the fluoride-resistant strain-containing biofilms not only benefited from its fluoride-resistant characteristics but also supported *C. albicans* growth in the presence of NaF, thus increasing the *C. albicans* CFU count from that in the fluoride-sensitive strain-containing cross-kingdom biofilm. The correlation between *S. mutans* and *C. albicans* could be synergetic, and accumulation of one in mixed-species biofilms could enhance the other (Chan et al., 2020; Khoury et al., 2020). However, this collaboration was insufficient for fluoride-sensitive *S. mutans*- and *C. albicans*-containing mixed-species biofilms to ameliorate the effects of NaF. Moreover, opportunistic cariogenic *S. mutans*, the main EPS producer, further promotes co-adhesion and cross-kingdom biofilm formation (Hwang et al., 2017). Thus, increased production of EPS by fluoride-resistant *S. mutans*-containing biofilms in the presence of NaF facilitates mixed-species biofilm formation. Additionally, bacteria-derived EPS enhances *C. albicans* resistance (Kim et al., 2018). The much more protected *C. albicans* in fluoride-resistant strain-containing biofilms in the presence of NaF could in return promote co-existence of these two microbes with biofilms, thus acting together against NaF with greater potential (Hwang et al., 2017; Khoury et al., 2020).

Important cariogenic virulence factors, such as EPS, are the main components of extracellular polymeric substances in cariogenic biofilms (Klein et al., 2015). It can facilitate bacterial binding, provide mechanical stability, protect microorganisms within biofilms, store energy and ions, and promote a highly acidic niche formation for demineralization (Klein et al., 2015). Though fungal β-glucan (encoded by *BGL2*) also contributes to the EPS matrix structure of the mixed-species biofilm model used in our study, bacteria-derived water-insoluble EPS (α-glucan) served as the main source for glucan in cross-kingdom biofilms (Falsetta et al., 2014; Lobo et al., 2019). In the presence of NaF, fluoride-resistant *S. mutans* biofilms generated more water-insoluble glucans than the wild-type biofilm (Supplementary Figure 4). Less water-insoluble glucans in single- or mixed-species biofilms in the presence of NaF than that in the respective control corroborated that NaF suppresses bacterial glucan production (Pandit et al., 2011). The increased total EPS content in fluoride-resistant *S. mutans*-containing cross-kingdom biofilms in the presence of NaF than that in the fluoride-sensitive *S. mutans*-containing biofilms might mainly be influenced by the presence of EPS production agents (fluoride-resistant *S. mutans*) and fungal accomplice, not primarily by alterations in the expression of related genes. The presence of *C. albicans* aggravates bacterial EPS production in addition to the *S. mutans*-produced EPS, and the impact of the bacterial count was much greater than that of the expression of EPS-related genes *gtfB* (water-insoluble glucan) and *gtfC* (water-insoluble and -soluble glucans). The upregulation of the expression of the β-glucan-encoding gene *BGL2* in *C. albicans* and bacterial glucan synthesis (*gtfB*; *gtfC*) and remodeling-related genes (*dexA*) in wild-type *S. mutans* in the presence of NaF might be due to their battle against NaF. The products of these genes facilitate biofilm formation and antimicrobial drug resistance (Kim et al., 2018). The expression of these genes in fluoride-resistant *S. mutans* was substantially different from that in fluoride-resistant *S. mutans*. *gtfB* expression was not upregulated significantly, while that of *gtfC* and *dexA* was downregulated in the presence of NaF than in its absence. This might be explained by the fact that fluoride resistance is another approach to resist NaF stress. *gtfD* is related to water-soluble glucans, and its expression was upregulated in both cross-kingdom biofilms in the presence of NaF, probably due to the bacterial energy required for further coping with fluoride stress. However, these specific molecular mechanisms require further study.

Despite being a substrate for EPS synthesis, sucrose was used by the core acid producer (bacteria) to produce acids. NaF inhibits acid production, another central cariogenic virulence factor (Pandit et al., 2011). In addition, NaF stress reduces microbial CFUs. Therefore, the pH of the supernatant within the mixed-species biofilms was higher than that of each control. In both types of *S. mutans*, NaF prominently suppressed the acid production gene *ldh*, and the acid tolerance gene *atpD* expression might be regulated in response to *ldh*. In addition, with the increased pH in the presence of NaF, expressions of *PHR1* (induced at pH ≥5) and *PHR2* (induced at pH ≤ 5) were prominently up- and down-regulated, respectively, in *C. albicans* (Chen et al., 2020). The fluoride-resistant single- or mixed-species biofilms still had low pH and produced acid in the presence of NaF (Figure 4; Supplementary Figure 5). Although *C. albicans* cannot utilize sucrose efficiently, *S. mutans* could cross-feed *C. albicans* by supporting sucrose catabolites, namely, glucose and fructose, to further enhance fungal growth and acid production (Ellepola et al., 2019). Low pH due to acid production not only induces EPS synthesis but also facilitates demineralization. The cross-kingdom biofilms used in our study have stronger demineralization ability than the corresponding single-species biofilm (Falsetta et al., 2014; Sampaio et al., 2019). Fluoride is the most valid anti-caries agent that not only has antimicrobial activity but also reduces demineralization and enhances remineralization (Ten Cate and Buzalaf, 2019). We tested whether fluoride-resistant strain-containing groups could demineralize in the presence of NaF and found that NaF reduced demineralization. Remarkably, fluoride-resistant *S. mutans* biofilms demineralized in the presence of 275 mg/L NaF. This outcome implies that fluoride-resistant *S. mutans* partially worsens the anti-caries activity of fluoride.

Signaling processes are involved in the interaction between *S. mutans* and *C. albicans*. The sigma factor sigX (encoded by *comX*), master regulator of competence development in *S. mutans*, is activated in the presence of *C. albicans* (Sztajer et al., 2014). We found that *comX* expression was significantly upregulated in the fluoride-sensitive, but not fluoride-resistant, strains with increased NaF concentration. Likewise, the expression of quorum sensing-associated genes *luxS* and *comDE*, a two-component system-related gene *vicR*, was prominently upregulated under NaF, especially for *luxS*. However, the expression of these genes was downregulated in the fluoride-resistant strain-containing groups. All these genes are essential for robust biofilm formation or promotes biofilm formation (Sun et al., 2021). The upregulation of the gene expressions might partly arise from the obvious inhibitory effect of NaF on bacterial biofilm formation; *S. mutans* upregulated these genes to facilitate biofilm formation in response, further helping cross-kingdom biofilms to tide over NaF stress. For *C. albicans*, the expression of hyphae wall protein gene *HWP1*, adhesins gene *ALS1*, and *ALS3* showed similar trends in response to NaF within cross-kingdom biofilms with or without wild-type biofilm, which might be attributed to fungal reaction to tide over NaF stress. *YWP1* expression was upregulated in fluoride-resistant strains in the presence of NaF, which might be due to the presence of NaF-sensitive *C. albicans* in these biofilms, which is involved in dissemination (Mayer et al., 2013).

In addition to above possible explanations, cell density results which displayed quite different cross-kingdom biofilm composition under NaF stress might also contribute to microbial gene expression alterations. One limitation of this study is that the transcription level of reference genes under different conditions or different stains is not monitored, which may affect the precision of microbial gene expression results. In addition, though our cross-kingdom biofilm model has demonstrated the adverse effects of fluoride-resistant *S. mutans in vitro*, further *in vivo* study is urgently needed. Moreover, more evidence about the prevalence of fluoride-resistant *S. mutans* or other opportunistic cariogenic microorganism in patients with caries or healthy people can support the influence of fluoride-resistant strain on caries development more convincingly. In addition, as fluoride-resistant strains may contain other irrelevant mutations, more fluoride-resistant strains, especially for clinically isolated strains, contribute significantly to figure out molecular mechanisms of fluoride resistance. In daily life, typical procedures to prevent dental caries wiping dental plaque biofilms include tooth brushing and dental flossing using fluoride, the most effective anti-caries agent known. When using fluoride for caries control, its side effects, such as fluoride-resistant strain development, should also be considered. Cocktail strategies that combine fluoride and other molecules with ecological effects may be a feasible option that adopting fluoride's positive effects on caries control, meanwhile avoiding its shortcomings. In addition, using fluoride-resistant probiotics or fluoride-resistant competitor of cariogenic bacteria maybe another strategy to combat fluoride-resistant cariogenic bacteria and its drawbacks.

## Conclusion

We demonstrated that fluoride-resistant *S. mutans* in the cross-kingdom biofilm supported *C. albicans* growth under fluorine stress and limited the *in vitro* anti-caries potential of fluorine by maintaining robust cross-kingdom biofilm formation and cariogenic virulence expression in the presence of NaF *in vitro*.

## Data availability statement

The original contributions presented in the study are included in the article/Supplementary material, further inquiries can be directed to the corresponding authors.

## Author contributions

YS: Funding acquisition, Investigation, Writing – original draft, Validation. YC: Investigation, Writing – original draft, Validation. QD: Formal analysis, Writing – original draft. JZ: Formal analysis, Writing – original draft. MX: Formal analysis, Writing – original draft. GZ: Formal analysis, Writing – original draft. WZ: Formal analysis, Writing – original draft. XZ: Formal analysis, Writing – original draft. LQ: Writing – review & editing, Supervision. YP: Funding acquisition, Writing – review & editing, Supervision. KZ: Funding acquisition, Writing – review & editing, Conceptualization.
