# Supplementary material for: Fluoride-resistant Streptococcus mutans within cross-kingdom biofilms support Candida albicans growth under fluoride and attenuate the in vitro anti-caries effect of fluorine
Source: Front Microbiol. 2024 Jul 5;15:1399525. doi: 10.3389/fmicb.2024.1399525 (PMC11257928; doi:10.3389/fmicb.2024.1399525)
Supplement: Supplementary file 1 [file Table_1.DOCX]

Supplementary Material

## Supplementary Materials and Methods

Growth curve

Overnight *S. mutans* and fluoride-resistant *S. mutans* cultures in Brain Heart Infusion (BHI) broth were used for bacterial proliferation. For bacterial growth curve, 10^6^ colony-forming unit (CFU)/mL of *S. mutans* or fluoride-resistant *S. mutans* was cultured in 96- well plates with 200 μL BHI broth in each well. The 96- well plates were incubated in a microplate reader (SpectraMax M5, Molecular Devices, USA) at 37 ℃ for 12 h and OD_600nm_ was recorded every 1 h (Sun et al., 2021).

.


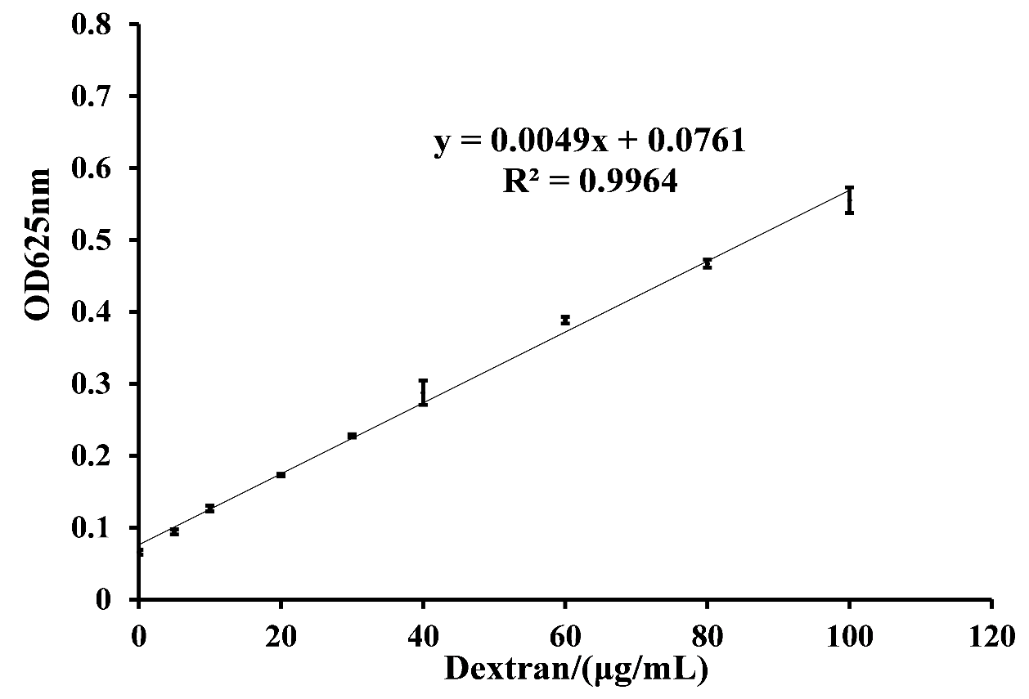


Supplementary Figure 1. Standard curve for water-insoluble polysaccharide detection.


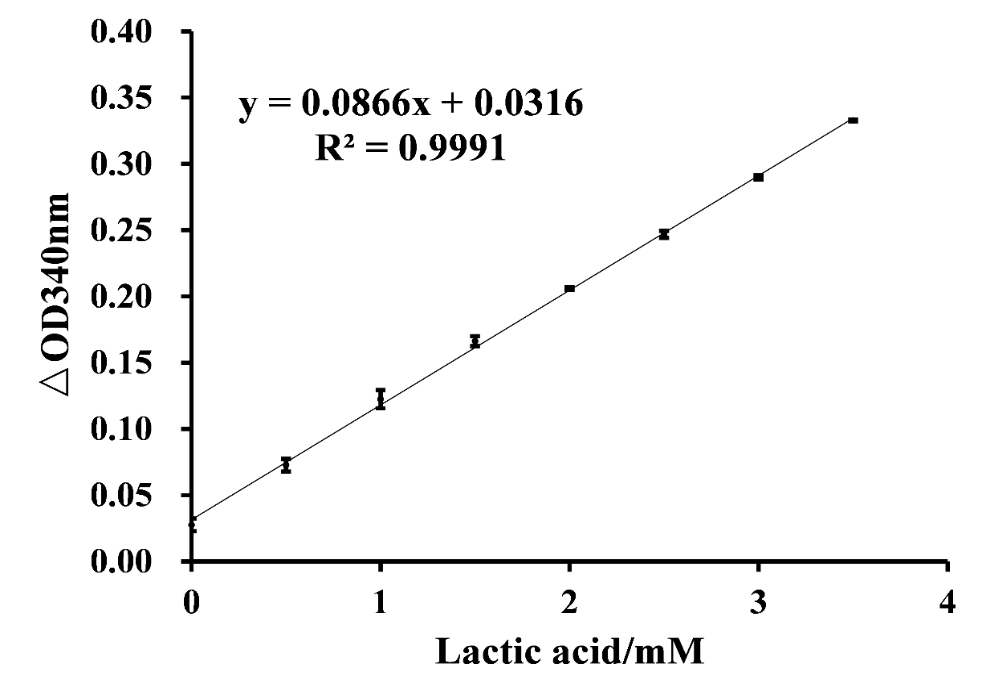


**Supplementary Figure 2.** Standard curve for lactic acid production.


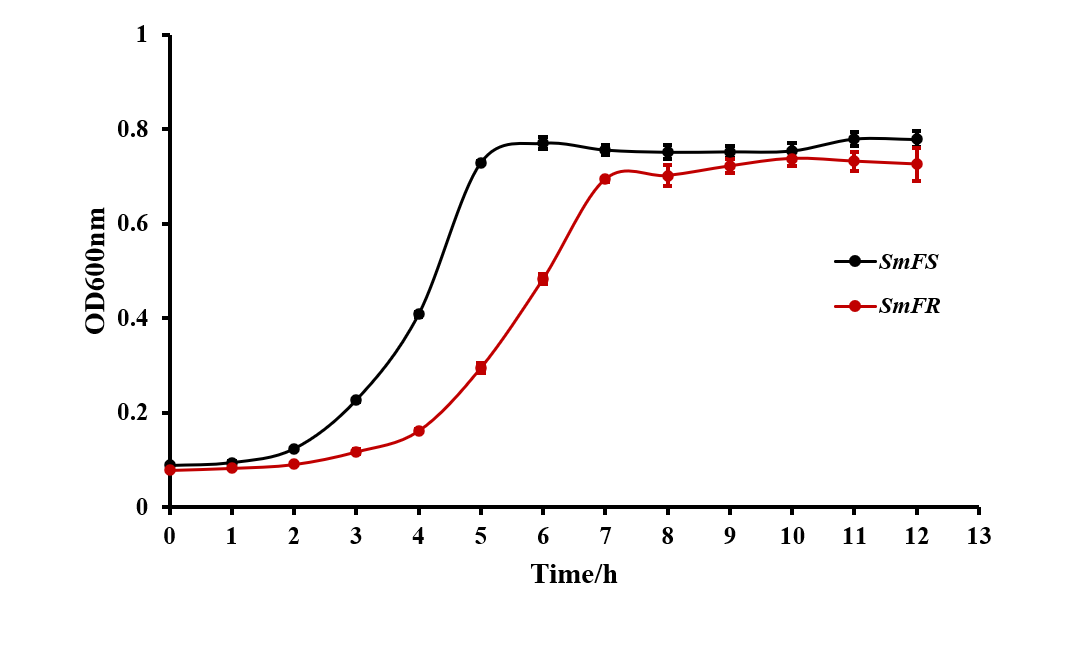


**Supplementary Figure 3.** Bacterial growth curve. *SmFS* represented fluoride-sensitive *S. mutans*, *SmFR* represented fluoride-resistant *S. mutans*. (Data were presented as mean ± standard deviation).


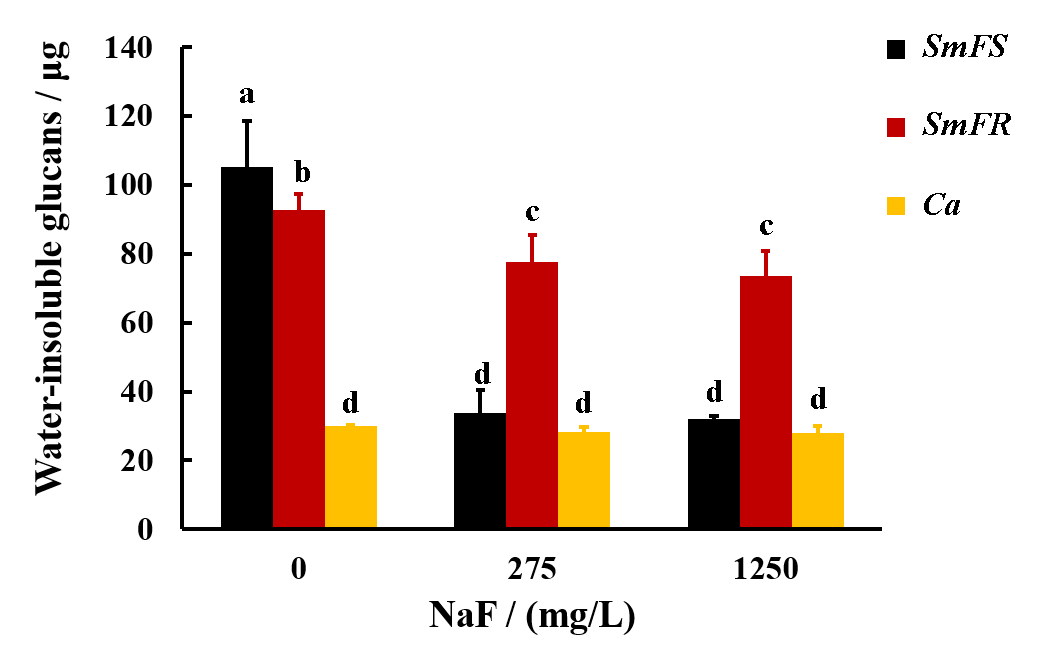


**Supplementary Figure 4.** Water-insoluble polysaccharide production of single species biofilm. *SmFS* represented fluoride-sensitive *S. mutans*, *SmFR* represented fluoride-resistant *S. mutans*, *Ca* represented *C. albicans*. (Data were presented as mean ± standard deviation. Values with dissimilar letters are identified as significantly different from other, P < 0.05).


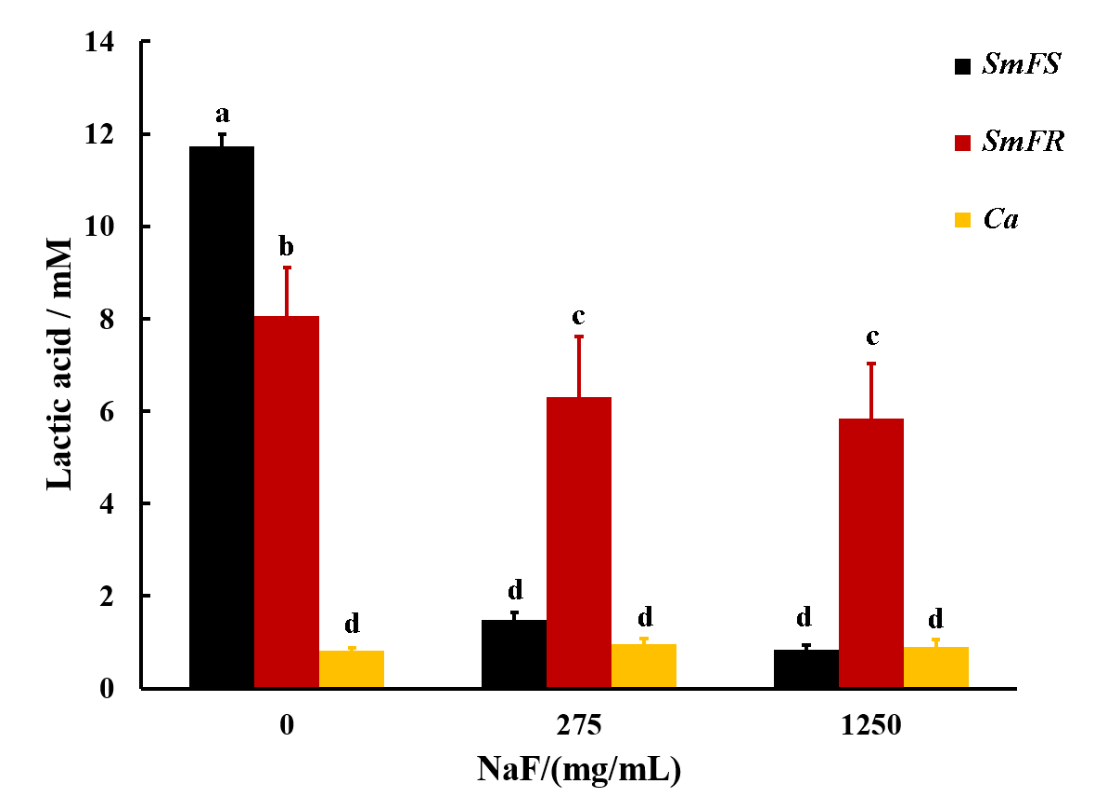


**Supplementary Figure 5.** Lactic acid production of single species biofilm. *SmFS* represented fluoride-sensitive *S. mutans*, *SmFR* represented fluoride-resistant *S. mutans*, *Ca* represented *C. albicans*. (Data were presented as mean ± standard deviation. Values with dissimilar letters are identified as significantly different from other, P < 0.05).

Supplementary Table 1 Oligonucleotide Primers Used in Fluorescent in situ hybridization (FISH)

| Probes | Nucelotide Sequence (5’-3’) | | Reference |
| --- | --- | --- | --- |
| *S. mutans*  *C. albicans* | Alexa Fluor 488-5’- ACTCCAGACTTTCCTGAC -3’  Alexa Fluor 555-5’- GCCAAGGCTTATACTCGCT -3’ | (Sun et al 2019)  (Kempf et al., 2010) | |

Supplementary Table 2 Oligonucleotide Primers Used in qRT- PCR

| Primers | | Nucelotide Sequence (5’-3’) | | Reference |
| --- | --- | --- | --- | --- |
| *18S*-f  *18S*-r  *HWP1*-f  *HWP1*-r  *YWP1*-f  *YWP1*-r  *ALS1*-f  *ALS1*-r  *ALS3*-f  *ALS3*-r  *BGL2*-f  *BGL2*-r  *PHR1*-f  *PHR1*-r  *PHR2*-f  *PHR2*-r  *16S*-f  *16S*-r  *gtfB*-f  *gtfB*-r  *gtfC*-f  *gtfC*-r  *gtfD*-f  *gtfD*-r  *ldh*-f  *ldh*-r  *atpD*-f  *atpD*-r  *dexA*-f  *dexA*-r  *luxS*-f  *luxS*-r  *comDE*-f  *comDE*-r  *comX*-f  *comX*-r  *vicR*-f  *vicR*-r | CACGACGGAGTTTCACAAGA  CGATGGAAGTTTGAGGCAAT  GCTCCTGCTCCTGAAATGAC  CTGGAGCAATTGGTGAGGTT  GCTACTGCTACTGGTGCTA  AACGGTGGTTTCTTGAC  GACTAGTGAACCAACAAATACCAGA  CCAGAAGAAACAGCAGGTGA  CAACTTGGGTTATTGAAACAAAAACA  AGAAACAGAAACCCAAGAACAACC  ATGGGTGATTTGGCTTTCAA  CAGCTGGACCAAGGTTTTGT  GGTTTGGTTCTGGTTGATGG  AGCAGCAGTTCCTGGACATT  CTCCTCCATTTCCAGAACCA  CGTCTGAATCAACCTTGTCG  CCTACGGGAGGCAGCAGTAG  CAACAGAGCTTTACGATCCGAAA  AGCAATGCAGCCAATCTACAAAT  ACGAACTTTGCCGTTATTGTCA  CTCAACCAACCGCCACTGTT  GGTTTAACGTCAAAATTAGCTGTATTAGC  ACAGCAGACAGCAGCCAAGA  ACTGGGTTTGCTGCGTTTG  AAAAACCAGGCGAAACTCGC  CTGAACGCGCATCAACATCA  TGTTGATGGTCTGGGTGAAA  TTTGACGGTCTCCGATAACC  TATTTTAGAGCAGGGCAATCG  AACCTCCAATAGCAGCATAAC  ACTGTTCCCCTTTTGGCTGTC  AACTTGCTTTGATGACTGTGGC  ACAATTCCTTGAGTTCCATCCAAG  TGGTCTGCTGCCTGTTGC  CGTCAGCAAGAAAGTCAGAAAC  ATACCGCCACTTGACAAACAG  CGTGTAAAAGCGCATCTTCG  AATGTTCACGCGTCATCACC | | (Feldman et al., 2016)  (Feldman et al., 2016)  (Feldman et al., 2016)  (Srivastava et al 2020)  (Feldman et al., 2016)  (Lobo et al., 2019)  (Lobo et al., 2019)  (Lobo et al., 2019)  (Sun et al., 2021)  (Sun et al., 2021)  (Sun et al., 2021)  (Sun et al., 2021)  (Sun et al., 2021)  (Sun et al., 2021)  (Bitoun et al., 2012)  (Sun et al., 2021)  (Sun et al., 2021)  (Sun et al., 2021)  (Sun et al., 2021) | |

**References**

Bitoun, J. P., Liao, S., Yao, X., Ahn, S. J., Isoda, R., Nguyen, A. H., Brady, L. J., Burne, R. A., Abranches, J., and Wen, Z. T. (2012). BrpA is involved in regulation of cell envelope stress responses in *Streptococcus mutans*. Appl Environ Microbiol 78(8), 2914-22. DOI: 10.1128/AEM.07823-11.

Feldman, M., Ginsburg, I., Al-Quntar, A., and Steinberg, D. (2016). Thiazolidinedione-8 Alters Symbiotic Relationship in *C. albicans*-*S. mutans* Dual Species Biofilm. *Front Microbiol* 7, 140. DOI: 10.3389/fmicb.2016.00140.

Kempf, V. A., Trebesius, K., & Autenrieth, I. B. (2000). Fluorescent In situ hybridization allows rapid identification of microorganisms in blood cultures. *J Clin Microbiol* 38(2), 830-8. DOI: 10.1128/JCM.38.2.830-838.2000.

Lobo, C. I. V., Rinaldi, T. B., Christiano, C. M. S., De Sales Leite, L., Barbugli, P. A., and Klein, M. I. (2019). Dual-species biofilms of *Streptococcus mutans* and *Candida albicans* exhibit more biomass and are mutually beneficial compared with single-species biofilms. *J Oral Microbiol* 11(1), 1581520. DOI: 10.1080/20002297.2019.1581520

Srivastava, N., Ellepola, K., Venkiteswaran, N., Chai, L. Y. A., Ohshima, T., and Seneviratne, C. J. (2020). Lactobacillus Plantarum 108 Inhibits *Streptococcus mutans* and *Candida albicans* Mixed-Species Biofilm Formation. Antibiotics (Basel) 9(8), 478. DOI: 10.3390/antibiotics9080478.

Sun, Y., Jiang, W., Zhang, M., Zhang, L., Shen, Y., Huang, S., Li, M., Qiu, W., Pan, Y., Zhou, L., and Zhang, K. (2021). The inhibitory effects of ficin on *Streptococcus mutans* biofilm formation. Biomed Res Int 2021, 6692328. DOI: 10.1155/2021/6692328.

Sun, Y., Pan, Y., Sun, Y., Li, M., Huang, S., Qiu, W., Tu, H., and Zhang, K. (2019). Effects of norspermidine on dual-species biofilms composed of *Streptococcus mutans* and *Streptococcus sanguinis*. *Biomed Res Int* 2019, 1950790. DOI: 10.1155/2019/1950790.
